# Meteorological Table

**Published:** 1860-11

**Authors:** J. G. Westmoreland

**Affiliations:** Smithsonian Institution, October, 1860, at Atlanta, Ga.


### METEOROLOGICAL OBSERVATIONS,

Taken by J. G. Westmoreland, M. D., for the Smithsonian Institution, October, 1860, at Atlanta, Ga., Latitude 33 deg. 45 min.—Longitude 7 deg. 30 min.—Height above the Sea, 1050 feet.

| Days of Month | BAROMETER. | | | THERMOMETER. | | | RAIN AND SNOW. | | CLOUDS. | | | WIND. | | |
|---|---|---|---|---|---|---|---|---|---|---|---|---|---|---|
| | 7 A.M. | 2 P.M. | 9 P.M. | 7 A.M. | 2 P.M. | 9 P.M. | Amt. Rn | In. Sn'w | 7 A.M. | 2 P.M. | 9 P.M. | 7 A.M. | 2 P.M. | 9 P.M. |
| 1 | 29.00 | 28.92 | 28.90 | 62 | 66 | 64 | | | 10 | 10 | 10 | E | E | E |
| 2 | 28.90 | 28.90 | 28.90 | 64 | 72 | 68 | .50 | | 10 | 10 | 10 | E | E | E |
| 3 | 28.88 | 28.85 | 28.80 | 68 | 68 | 68 | .50 | | 10 | 10 | 10 | E | E | E |
| 4 | 28.80 | 28.82 | 28.85 | 68 | 76 | 68 | | | 10 | 5 | 5 | E | S E | S E |
| 5 | 28.85 | 28.85 | 28.85 | 68 | 78 | 70 | | | 10 | 5 | 5 | S E | S E | S E |
| 6 | 28.85 | 28.85 | 28.80 | 70 | 82 | 74 | 4.50 | | 5 | 5 | 10 | S E | S E | S E |
| 7 | 28.80 | 28.80 | 28.75 | 66 | 78 | 70 | .25 | | 10 | 5 | 10 | S E | S E | S E |
| 8 | 28.70 | 28.70 | 28.64 | 68 | 80 | 64 | | | 10 | 5 | 5 | S E | S W | W |
| 9 | 28.62 | 28.65 | 28.65 | 50 | 62 | 56 | | | 0 | 0 | 0 | W | W | W |
| 10 | 28.65 | 28.70 | 28.65 | 50 | 67 | 60 | | | 5 | 0 | 5 | S E | S E | S E |
| 11 | 28.65 | 28.70 | 28.70 | 58 | 62 | 52 | | | 5 | 10 | 5 | S E | S E | S E |
| 12 | 28.70 | 28.70 | 28.70 | 46 | 60 | 50 | | | 0 | 0 | 0 | W | W | W |
| 13 | 28.70 | 28.70 | 28.75 | 42 | 46 | 42 | | | 5 | 5 | 5 | N W | N W | N W |
| 14 | 28.75 | 28.80 | 28.85 | 38 | 52 | 48 | | | 0 | 0 | 0 | N W | N W | N W |
| 15 | 28.90 | 28.95 | 28.90 | 36 | 56 | 40 | | | 0 | 0 | 0 | N W | N W | N W |
| 16 | 28.90 | 28.95 | 28.90 | 36 | 60 | 50 | | | 0 | 0 | 0 | N W | N W | N W |
| 17 | 28.80 | 28.80 | 28.80 | 46 | 68 | 60 | | | 0 | 0 | 0 | N W | N W | N W |
| 18 | 28.70 | 28.70 | 28.60 | 48 | 60 | 50 | 1.00 | | 0 | 5 | 10 | N E | N E | E |
| 19 | 28.50 | 28.45 | 28.45 | 48 | 48 | 48 | 1.25 | | 10 | 10 | 10 | E | E | E |
| 20 | 28.40 | 28.48 | 28.50 | 48 | 50 | 48 | | | 10 | 10 | 0 | E | E | N W |
| 21 | 28.65 | 28.70 | 28.75 | 48 | 56 | 50 | | | 10 | 5 | 5 | N W | N W | N W |
| 22 | 28.75 | 28.80 | 28.80 | 50 | 65 | 52 | | | 5 | 0 | 0 | N W | N W | N W |
| 23 | 28.75 | 28.80 | 28.75 | 50 | 68 | 52 | | | 0 | 0 | 0 | N W | W | W |
| 24 | 28.75 | 28.80 | 28.75 | 50 | 70 | 60 | | | 0 | 0 | 0 | W | W | W |
| 25 | 28.75 | 28.80 | 28.80 | 50 | 78 | 60 | | | 0 | 0 | 0 | W | W | W |
| 26 | 28.85 | 28.85 | 28.85 | 58 | 72 | 58 | | | 0 | 5 | 5 | W | S | S W |
| 27 | 28.85 | 28.90 | 28.90 | 58 | 72 | 60 | | | 5 | 5 | 5 | S W | S W | S W |
| 28 | 28.85 | 28.80 | 28.75 | 62 | 68 | 58 | 1.25 | | 10 | 10 | 10 | S W | S W | S W |
| 29 | 28.80 | 28.85 | 28.80 | 56 | 66 | 58 | | | 0 | 0 | 0 | W | W | W |
| 30 | 28.75 | 28.85 | 28.80 | 48 | 66 | 54 | | | 0 | 0 | 0 | W | W | W |
| 31 | 28.80 | 28.80 | 28.80 | 52 | 70 | 60 | | | 5 | 5 | 5 | W | W | W |

